# A Specific and Sensitive Enzymatic Assay for the Quantitation of L-Proline

**DOI:** 10.3389/fpls.2020.582026

**Published:** 2020-10-22

**Authors:** Giuseppe Forlani, Dietmar Funck

**Affiliations:** ^1^Department of Life Science and Biotechnology, University of Ferrara, Ferrara, Italy; ^2^Laboratory of Plant Physiology and Biochemistry, Department of Biology, University of Konstanz, Konstanz, Germany

**Keywords:** proline measurement, chemical assay, enzymatic assay, specificity, sensitivity

## Abstract

Because proline accumulates rapidly in response to several stress conditions such as drought and excess salt, increased intracellular levels of free proline are considered a hallmark of adaptive reactions in plants, particularly in response to water stress. Proline quantitation is easily achievable by reaction with ninhydrin, since under acidic conditions peculiar red or yellow reaction products form with this unique cyclic amino acid. However, little attention has been paid to date to cross-reaction of ninhydrin with other amino acids at high levels, or with structurally related compounds that may also be present at significant concentrations in plant tissues, possibly leading to proline overestimation. *In vitro* at high pH values, δ^1^-pyrroline-5-carboxylate reductase, the enzyme catalyzing the second and last step in proline synthesis from glutamate, was early found to catalyze the reverse oxidation of proline with the concomitant reduction of NAD(P)^+^ to NAD(P)H. Here we characterized this reverse reaction using recombinant enzymes from *Arabidopsis thaliana* and *Oryza sativa*, and demonstrated its utility for the specific quantification of L-proline. By optimizing the reaction conditions, fast, easy, and reproducible measurement of L-proline concentration was achieved, with similar sensitivity but higher specificity than the commonly used ninhydrin methods.

## Introduction

Water stress tolerance is in the focus of many research projects and also a major goal for plant breeding to secure crop productivity in the face of ongoing climate change (Ahanger et al., 2017). Accumulation of free proline (this term referring to the L-isomer, if not specified otherwise) is a common response of plants to diverse stress factors, mainly drought and high salinity, but also to cold or pathogen attack (Hayat et al., 2012). Therefore, rapid and cost-efficient assays to determine tissue proline content are useful to monitor plant stress responses or to assess stress tolerance. Chinard (1952) reported that at approximately pH 1.0 ninhydrin reacts with only a few amino acids, and that this reaction can be used to quantify proline, ornithine, lysine, and hydroxylysine in pure solution. Later on, a modification of this method was described in which lysine and hydroxylysine do not react (Bates et al., 1973), establishing a quick assay for proline in plant extracts that has become the most widely adopted reference method (cited by more than 12,000 articles) and is used with minor modifications until today. This assay takes advantage of the formation of a red, hydrophobic reaction product from proline and ninhydrin at very low pH and high temperature (100°C), while all other proteinogenic amino acids produce little or no color. Extraction of the reaction product with toluene avoids interference from hydrophilic red plant pigments like anthocyanins, but constitutes an additional, potentially harmful handling step that produces toxic waste. Moreover ornithine, an intermediate in the synthesis and degradation of arginine (Winter et al., 2015), reacts under these conditions with the same efficiency, and some other amino acids like glutamine show 1–2% color yield of equivalent amounts of proline (Bates et al., 1973). The interference of these ninhydrin-positive substances is negligible in those cases in which proline content in plant tissues is found to increase hundredfold in response to hyperosmotic stress (e.g., Handa et al., 1983; Binzel et al., 1987; Santos-Díaz and Ochoa-Alejo, 1994). However, during recent years increasing evidence has been reported that suggests a more faceted protective role of intracellular free proline (Sharma et al., 2011; Shinde et al., 2016; Signorelli, 2016), and even slight increases of its intracellular content, well below those required for effective osmotic compensation, were found beneficial under stress. The exact mechanisms underlying these positive effects are still a matter of debate (Forlani et al., 2019b), and some data also suggest that an increase in proline metabolic rates, more than its absolute concentration, may help the cell to counteract abiotic stress conditions (Kavi Kishor and Sreenivasulu, 2014; Forlani et al., 2019a). To allow a deeper insight in these metabolic plant responses, there would consequently be a need of methods able to reliably detect even minor variations in proline levels.

In another variant of the acid ninhydrin assay, in which the reaction is carried out at lower temperature (50°C) and less acidic conditions (about pH 3.0), proline yields a peculiar yellow product, whereas most primary amines – like the other proteinogenic amino acids and ornithine – produce red to purple adducts (Williams and Frank, 1975). Conveniently, the reaction mixture can be read as it is, without the need of extracting the chromophore with an organic solvent. However, this protocol has been specifically developed for the quantitation of pure δ^1^-pyrroline-5-carboxylic acid (P5C), an intermediate in all the plant routes for proline biosynthesis and catabolism (Trovato et al., 2019), and its specificity when used for proline determination has not been thoroughly investigated. Therefore, in this case as well, standard solutions for calibration should comprise a mixture of amino acids similar to their abundance in the plant extracts, and reaction blanks without ninhydrin are required to subtract various plant compounds able to absorb at the same wavelength.

The limitations inherent to both these established protocols could be overcome by the availability of a robust and specific enzymatic assay for proline quantification. Proline dehydrogenase (ProDH, EC 1.5.5.2), the enzyme that oxidizes proline to P5C in plant mitochondria, is attached to the inner mitochondrial matrix and is very difficult to purify. Moreover, it is believed to transfer electrons directly to quinones of the respiratory electron transfer chain (Cabassa-Hourton et al., 2016), and is unable to catalyze *in vitro* the reduction of NAD(P)^+^ (Lanfranchi et al., manuscript in preparation). The opposite reaction, *i.e.*, the NAD(P)H-dependent reduction of P5C to proline, is catalyzed *in vivo* by P5C reductase (P5CR, EC 1.5.1.2; Forlani et al., 2015b). During early purification attempts, a single protein was found to possess both P5CR and proline dehydrogenase activities (Rena and Splittstoesser, 1975), and later on a purified P5CR was shown to catalyze the reverse reaction at alkaline pH (Szoke et al., 1992). A protocol to assay P5CR following the proline-dependent reduction of NAD^+^ at pH 10 was then reported (Chilson et al., 1992) and, because P5C is not commercially available, in many subsequent papers this reaction (or even the oxidation of proline analogs such as thiaproline) was used to assay the enzyme (e.g., Nocek et al., 2005; Meng et al., 2006), though it does not represent a physiological feature. We have previously described P5CRs from *Arabidopsis thaliana*, *Oryza sativa*, and *Medicago truncatula* as very stable enzymes that can easily be stored at 4°C over prolonged periods with negligible loss of activity (Funck et al., 2012; Giberti et al., 2014; Forlani et al., 2015a; Ruszkowski et al., 2015). Based on these premises, we established the reverse reaction of P5CR under optimized conditions as a simple, specific and reliable method for proline quantification.

## Materials and Methods

### Heterologous Expression and Affinity Purification of Plant P5CRs

Cloning and functional characterization of Arabidopsis and rice P5CRs have been previously described (Giberti et al., 2014; Forlani et al., 2015a). Overexpression was carried out in *Escherichia coli* BL21 (DE3) pLysS cells. The bacteria were grown at 24 °C in 2-L Erlenmeyer flasks with shaking at 100 rpm in 400 mL LB medium supplemented with 100 μg mL^–1^ ampicillin and 50 μg mL^–1^ chloramphenicol. At an OD_600_ of 0.5, expression of P5CR was induced with 1 mM isopropyl-β-D-thiogalactopyranoside. The culture was further incubated for 6 h, then cells were harvested by centrifugation at 3,000 *g* for 10 min and pellets were weighed and frozen at −20 °C. The samples (about 1 g) were thawed and extracted in an ice-cold mortar with 2 g g^–1^ alumina powder, then the homogenate was resuspended in 25 mL g^–1^ of extraction buffer (50 mM sodium phosphate, pH 7.5, 200 mM NaCl and 20 mM imidazole) containing 0.5 mM DTT. Cell debris was pelleted by centrifugation at 12,000 *g* for 10 min at 4 °C. The supernatant was applied at a constant flow of 10 mL h^–1^ to a 0.1 mL His SpinTrap column (GE Healthcare). After binding, the column was washed with 5 mL of extraction buffer and eluted stepwise with 3-mL aliquots of buffer containing increasing concentrations of imidazole (50, 100, 200, 350, and 500 mM), while collecting 1-mL fractions. Both Arabidopsis and rice P5CR eluted reproducibly and quantitatively at about 350 mM imidazole (Supplementary Figure S1). The His_6_-tag was not cleaved, as it has been shown to have no influence on the functional properties of the enzymes (Giberti et al., 2014; Forlani et al., 2015a). Protein concentration was determined by the method of Bradford (1976), using bovine serum albumin as the standard. Homogeneity of purified preparations was checked by discontinuous SDS-PAGE (12% acrylamide separating gel). Enzyme preparations were filter-sterilized (0.2 μm) and stored at 4 ± 2°C in the dark.

### P5CR Assays

Assays were performed in either 1 cm/0.2 cm path cuvettes (UVette; Eppendorf, Milan, Italy) in a final volume of 1 mL, or 96-microwell plates in a final volume of 0.2 mL. In the former case, OD_340_ was determined with a Novaspec plus spectrophotometer (Amersham Biosciences, Milan, Italy) equipped with a Peltier device set to 37°C and a UVette adaptor. In the latter case, the plate was equilibrated at 37°C prior to enzyme addition, and absorbance was measured using a Ledetect plate reader (Labexim, Lengau, Austria) equipped with a LED plugin at 340 nm. Each sample was carried out in triplicate (technical replications). Each determination was repeated with at least three different enzyme preparations (biological replications), obtaining almost identical patterns. Presented data refer to a single enzyme preparation, and are means ± SE over technical replicates. Linear and non-linear regressions of data were computed using Prism 6 for Windows, version 6.03 (GraphPad Software, San Diego, CA, United States).

#### Forward Assay

The physiological activity of P5CR was routinely measured at pH 7.5 as the P5C-dependent oxidation of NAD(P)H. P5C was synthesized by the periodate oxidation of δ-*allo*-hydroxylysine (Sigma H0377), purified by cation exchange chromatography on a 200–400 mesh Dowex AG50W-X4 column, and quantified as described (Williams and Frank, 1975). P5C solutions in 1 M HCl were stored at 4°C in the dark, and brought to neutral pH just before the assay using proper aliquots of a 1 M Tris base solution. Unless otherwise specified, the assay mixture contained 2 mM DL-P5C and 0.4 mM of either NADH or NADPH in 50 mM Tris-HCl buffer, pH 7.5. Parallel blanks were performed in which P5C had been omitted.

#### Reverse Assay

The reverse activity of P5CR was routinely measured at pH 10.2 as the proline-dependent reduction of NAD(P)^+^. Unless otherwise specified, the assay mixture contained 10 mM L-proline and 5 mM of either NAD^+^ or NADP^+^ in 100 mM glycine-NaOH buffer, pH 10.2. Parallel blanks were performed in which proline had been omitted. Kinetic analyses were performed by varying a single substrate while maintaining the invariable substrate at 10 mM NADP^+^ or 20 mM NAD^+^, and 50 mM (with NAD^+^) or 5 mM (with NADP^+^) proline in the case of *O. sativa* P5CR; at 3 mM NADP^+^ or 15 mM NAD^+^, and 2 mM proline in the case of the enzyme from *A. thaliana*. *K*_*M*_ and *V*_*max*_ values and their confidence intervals were computed using the corresponding functions in the Prism 6 software.

### Ninhydrin Assays

Assays were performed using either the protocols described by Bates et al. (1973) or Williams and Frank (1975). No changes were introduced, but volumes were reduced while maintaining proportion and composition of the solutions. Solutions of pure proline in water were used to calibrate the assays. In all cases samples were carried out in triplication, and mean values ± SE are reported. Each experiment was repeated twice.

#### Bates Protocol

Ninhydrin was dissolved at 25 mg mL^–1^ in 60% (v/v) acetic acid – 13.8% (w/v) phosphoric acid. Samples (100 μL) were mixed with the same volume of both ninhydrin solution and glacial acetic acid, and incubated at 100°C for 60 min. After cooling, the formed chromophore was extracted with 200 μL toluene by vortexing a few times. Following centrifugation at 10,000 *g* for 3 min, the organic phase was read at 520 nm in quartz or PMMA cuvettes using an Ultrospec 1100 pro spectrophotometer (Amersham Biosciences).

#### Williams and Frank Protocol

Ninhydrin was dissolved at 1.5 mg mL^–1^ in glacial acetic acid. Samples (15 μL) were sequentially mixed with 15 μL of 3 M Na acetate and 200 μL ninhydrin solution, and immediately read in PS cuvettes with the Ultrospec 1100 pro spectrophotometer, or in 96-microwell plates with the Ledetect plate reader equipped with LED plugins at 352, 520 or 540 nm. After incubation at 50°C for 12.5 min, samples were cooled to room temperature and read again. The difference of absorbance between final reading and time-zero value was considered.

### Cell Culture, Growth Conditions and Amino Acid Extraction

*Nicotiana plumbaginifolia* Viviani suspension cultured cells were grown heterotrophycally at 24°C in the dark, as described previously (Forlani et al., 1996). Cultures were treated with 175 mM NaCl three days after subculturing, when cells had entered the exponential phase of growth. At increasing time after the treatment, culture aliquots were withdrawn, and cells were harvested on nylon filters (2 μm) under vacuum. Cells were split into two samples, which were resuspended with 2 mL g^–1^ of either 3% (w/v) 5-sulfosalicylic acid solution or 100 mM glycine-NaOH buffer, pH 10.5, and extracted with a teflon-in-glass Potter homogenizer with 20 strokes. The homogenates were centrifuged 3 min at 12,000 *g*, and the supernatants were stored at −20°C until analyzed. Just before the analysis, extracts in glycine-NaOH buffer were treated 5 min at 95°C to completely inactivate endogenous enzymes, and then further centrifuged.

## Results

### Ninhydrin-Based Methods Can Significantly Overestimate Proline Content

**FIGURE 1:**
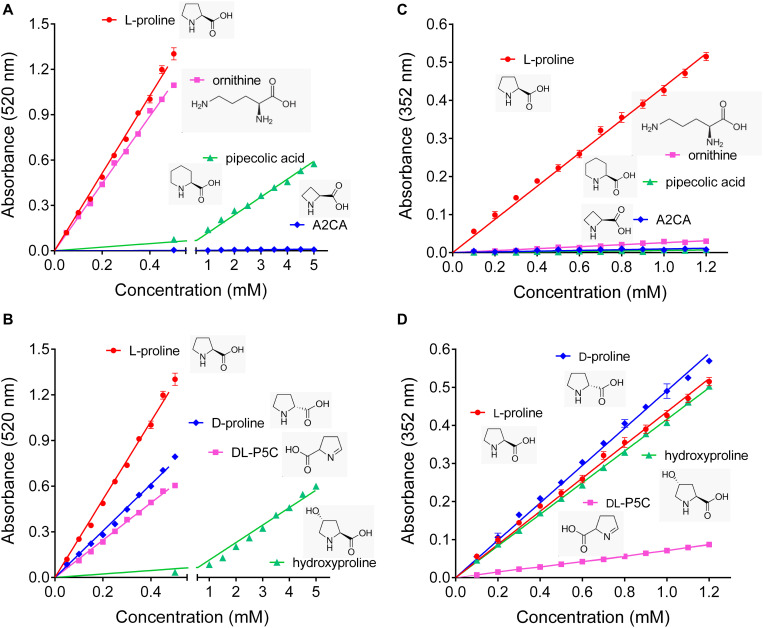
Specificity of the established ninhydrin-based methods for proline quantification. **(A,B)** In the assay developed by Bates et al. (1973), ninhydrin reacts also with ornithine, D-proline and DL-pyrroline-5-carboxylate (P5C), while pipecolate and hydroxyproline produce only low absorbances, and azetidin-2-carboxylate (A2CA) is undetectable. **(C,D)** The modified assay by Williams and Frank (1975) shows less interference by ornithine, pipecolate and DL-P5C, but the absorbance yield with D-proline and hydroxyproline is almost the same as for proline. All data are means ± SE over four replicates.

To get an estimate of the errors introduced by the commonly used ninhydrin assays for proline quantification, their specificity and possible interference from other amino acids were re-investigated. We confirmed that the Bates assay detects ornithine with almost the same sensitivity as proline, and also pipecolate, which differs from proline by an additional C-atom in the ring, was detected, although with a sensitivity one order of magnitude lower (Figure 1A). The four-atom ring analog azetidin-2-carboxylate (A2CA) was not detected. D-proline and DL-P5C produced about 60% and 50% of the absorbance at 520 nm compared to equivalent concentrations of L-proline, while hydroxyproline reacted with similar efficiency as pipecolate (Figure 1B). The modified assay by Williams and Frank showed a somehow higher specificity, but equally suffered from some interferences. Ornithine produced only 5% of the absorption at 352 nm compared to equimolar concentrations of proline, and the absorbance obtained with the same levels of pipecolate and A2CA was negligible (Figure 1C). However, P5C gave rise to 10% of the optical density obtained with proline, and D-proline and hydroxyproline produced a yellow adduct with nearly the same absorbance intensities as L-proline (Figure 1D).

**FIGURE 2:**
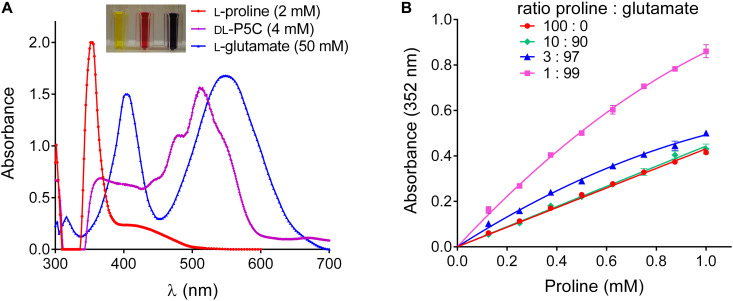
Overestimation of proline content by the Williams and Frank assay in samples containing high levels of other amino acids. **(A)** Absorption spectra of the reaction products of proline, P5C and glutamate under the conditions described by Williams and Frank (1975) show different intensities and absorption maxima, but all contribute to OD at 352 nm, which is used to quantify proline. **(B)** In extracts in which proline represents a minor component of the pool of free amino acids, this overlap leads to a significant overestimation of its content. Data in panel **(B)** refer to samples in which proline has been analyzed alone, or in combination with glutamate, as indicated, and are means ± SE over four replicates.

Visually, the products formed by the Williams and Frank assay from proline, P5C and primary amino acids were clearly different, yielding yellow, red or purple color, respectively (Figure 2A). However, when we analyzed the absorption spectra, it became evident that also the products formed from P5C and glutamate absorb – even if slightly – at 352 nm, the wavelength that is typically used to quantify proline. Changing the measuring wavelength would strongly impair the sensitivity of the assay without much gain in specificity. Because the reaction of proline with ninhydrin produces color with much higher efficiency, the error produced by the contribution of primary amino acids is negligible when proline constitutes 10% or more of the total amino acids. However, when proline was only 1% of the total amino acids, the absorption at 352 nm was approximately twice as high as for pure proline (Figure 2B). Since the amino acid composition of an unknown sample is virtually impossible to predict, none of the two ninhydrin-based assays is satisfactory to obtain a precise measure of proline content in plant tissues, and a novel method would be required.

### At Alkaline pH, Plant P5CR Mediates Proline-Dependent NAD(P)^+^ Reduction

**FIGURE 3:**
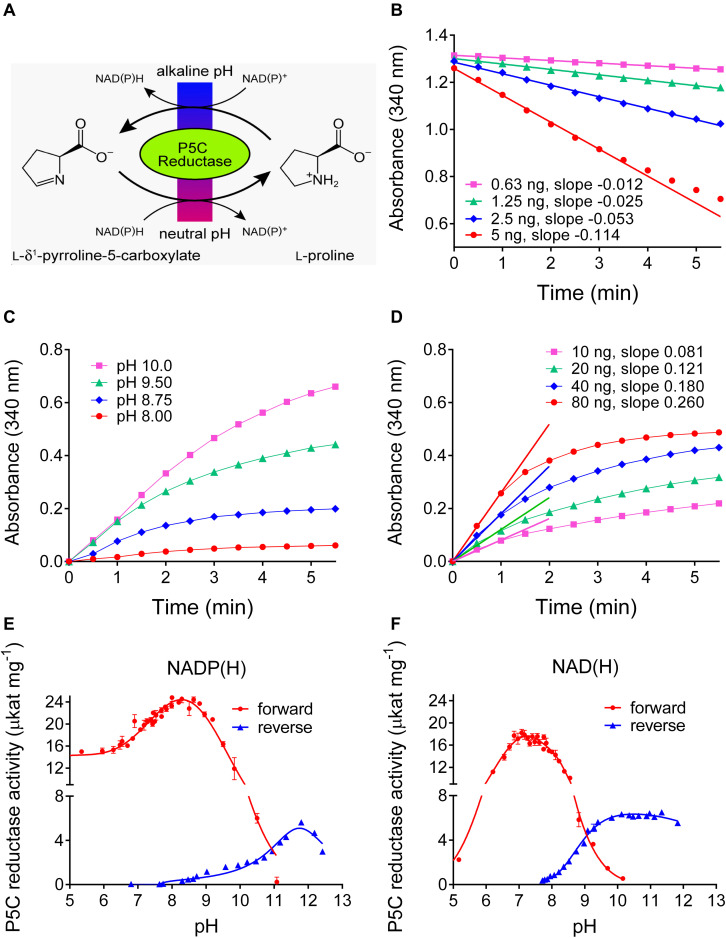
Forward and reverse reactions of Arabidopsis P5CR. **(A)** *In vivo* the enzyme catalyzes the reduction of P5C to proline, using either NADH or NADPH as the electron donor. However, at alkaline pH *in vitro*, also the reverse reaction takes place. **(B)** At pH 7.5, the P5C-dependent oxidation of NADH is linear over long times, and proportional to the amount of enzyme. **(C)** Proline-dependent NAD^+^ reduction by 100 ng P5CR is detectable only at pH ≥ 8.0, is strongly dependent on the pH of the assay mixture, and rapidly reaches a plateau. **(D)** Even at pH 10.2, no clear linear phase of NAD^+^ reduction is evident and the estimated slopes are not proportional to the amount of enzyme and less steep compared to the forward reaction. Same conditions as in panel **(C)** were used, but pH 10.2. **(E,F)** The pH-activity relationship of the forward and the reverse reaction was characterized by incubating 100 ng P5CR in 50 mM Hepes mixed with increasing concentrations of KOH, and using either NADP(H) **(E)** or NAD(H) **(F)** as cofactor. The actual pH value in each sample was measured at the end of the reaction with a microelectrode. Values refer to the initial, quasi-linear rates, and are the mean ± SE over three replicates. Similar results were obtained with rice P5CR.

Plants produce a plethora of metabolites, thus the specificity of any chemistry-based colorimetric assay is difficult to predict or confirm. An enzyme-based assay is supposed to have a much narrower range of substrates, and appears as a possible solution for a specific assay for L-proline. Isolation and purification of ProDH from plants has not been reported so far, but an alternative is represented by the reverse reaction of P5CR at high pH (Figure 3). We purified His-tagged recombinant Arabidopsis and rice P5CRs after expression in *E. coli* by Ni-affinity chromatography (Supplementary Figure S1). Starting from 400 mL of bacterial culture, about 400–500 and 800–900 μg of purified protein were obtained, respectively. At pH 7.5 *in vitro*, the conversion of P5C to proline by P5CR appeared as a virtually unidirectional reaction, which proceeded without product inhibition until the NAD(P)H/NAD(P)^+^ ratio became unfavorable (Figure 3B) (Giberti et al., 2014; Forlani et al., 2015a). However, under more alkaline conditions the reaction was found to proceed also in the reverse direction, and kinetic analysis revealed that both the initial rate of NAD^+^ reduction and the NADH level at equilibrium were increasing between pH 8 and 10 (Figure 3C). However, even at pH 10.2 the resulting rates were found significantly lower than those of the physiological reaction at neutral pH, were not proportional to the amount of enzyme, and rapidly lost linearity, yielding only semiquantitative results (Figure 3D).

To obtain a comprehensive picture of the pH dependence of the reactions catalyzed by plant P5CR, the initial rates of the forward and the reverse reaction were measured over a wide range of pH values, using either NAD(P)^+^ or NAD(P)H as cofactors (Figures 3E,F). With NADH as the electron donor, the forward reaction became undetectable above pH 10.2, and with NADPH above pH 11.0. The reverse reaction with NADP^+^ had a rather narrow pH optimum around pH 11.8, whereas with NAD^+^ as electron acceptor, a broad optimum between pH 9.5 and 12 was observed.

Based on the pH-dependence of the initial reaction rates, we reasoned that the reverse P5CR reaction could be employed for a quantitative assay for L-proline. A thorough kinetic analysis of the reverse reactions of Arabidopsis and rice P5CRs was performed to determine the constraints for the optimal assay conditions (Supplementary Figure S2). The results, summarized in Table 1, showed that both enzymes have a higher affinity for NADP^+^ than for NAD^+^, and that the affinity for proline is also higher when NADP^+^ is used as the electron acceptor. However, with NADP^+^ as the cofactor, proline concentrations higher than 2–3 mM in the case of the enzyme from Arabidopsis, or higher than 5–7 mM for that from rice, were found inhibitory (data not shown). Moreover, with NAD^+^ as cofactor, both enzymes showed much higher *V*_*max*_ values, and reached maximal catalytic efficiencies around 25 μkat (mg protein)^–1^. We concluded that both enzymes could be used for quantitative assays of proline in the micromolar range when a saturating concentration of NAD^+^ was provided.

**TABLE 1:** Kinetic properties of the reverse reaction of P5CR.

	Rice P5CR	Arabidopsis P5CR	
*V*_*max*_ (NAD^+^)	25.64 ± 0.90	33.92 ± 0.45	μkat (mg protein)^–1^
*V*_*max*_ (Pro, with NAD^+^ as the co-substrate)	21.00 ± 0.80	25.57 ± 0.70	μkat (mg protein) ^–1^
*V*_*max*_ (NADP^+^)	0.376 ± 0.003	3.94 ± 0.66	μkat (mg protein) ^–1^
*V*_*max*_ (Pro, with NADP^+^ as the co-substrate)	0.387 ± 0.005	3.77 ± 0.12	μkat (mg protein) ^–1^
K_M_(app) for L-Pro (NAD^+^)	4.86 ± 0.49	2.38 ± 0.18	mM
K_M_(app) for L-Pro (NADP^+^)	0.223 ± 0.016	0.378 ± 0.038	mM
K_M_(app) for NAD^+^	15.01 ± 1.05	7.17 ± 0.21	mM
K_M_(app) for NADP^+^	0.321 ± 0.016	0.517 ± 0.034	mM

*Data used for calculating kinetic constants are reported in Supplementary Figure S2.*

### At pH 10.5 ± 0.1 the Reverse P5CR Reaction Allows Reliable Quantitation of L-Proline

**FIGURE 4:**
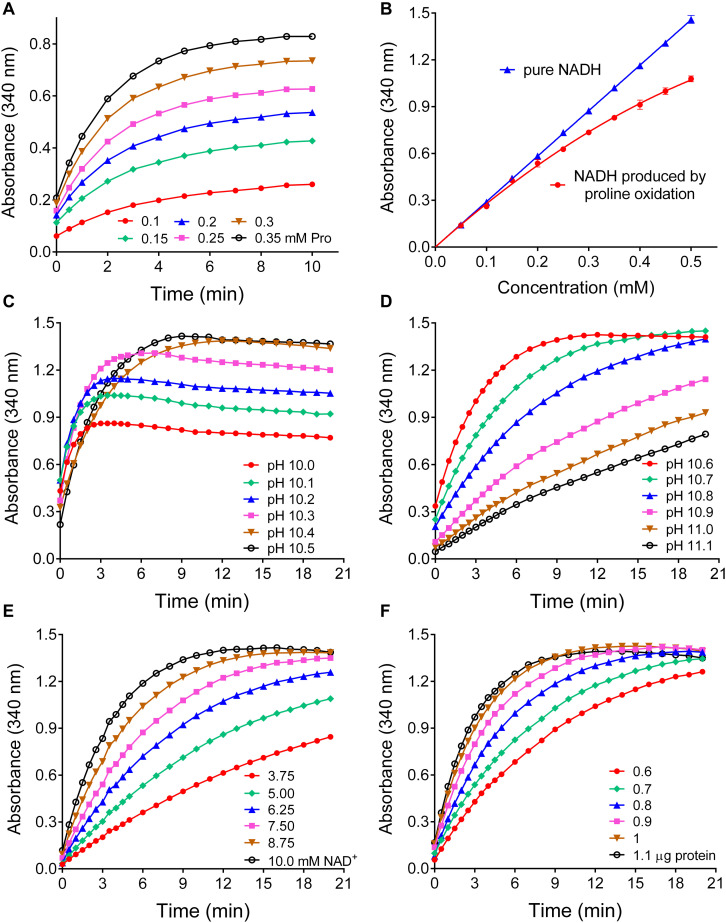
Effects of various parameters on the sensitivity and the linearity of a proline assay based on the reverse reaction of P5CR. **(A)** When increasing concentrations of proline were incubated at pH 10.2 in the presence of 10 mM NAD^+^ and a large excess of P5CR (1 μg protein), the production of NADH reached a proline concentration-dependent plateau in about 10 min. **(B)** Under these conditions, the reverse P5CR reaction yielded sub-stoichiometric concentrations of NADH above 150 μM proline. **(C)** When the pH of the reaction mixture was gradually varied, the optimal yield of NADH from 0.5 mM proline was reached at pH values exceeding 10.3. **(D)** Above pH 10.6 the time required for the completion of the reaction increased gradually. **(E,F)** Lowering NAD^+^ **(E)** or enzyme concentration **(F)** at pH 10.5 reduced the reaction velocity with little effect on the final NADH concentration. Presented results are means ± SE over at least three replicates, and were obtained with P5CR from rice. The experiment was repeated twice with the same results, and very similar patterns were found also with the enzyme from Arabidopsis.

Based on the kinetic data, a large excess of rice P5CR was incubated at pH 10.2 with a nearly saturating NAD^+^ concentration and increasing levels of proline in the micromolar range. Under this condition, NADH production reached a plateau within 10 min, and its level was proportional to the initial proline concentration (Figure 4A). However, at concentrations exceeding 150 μM proline, the reaction appeared not to proceed to completion, and less than equimolar concentrations of NADH were produced (Figure 4B). Because the results presented in Figure 3 imply that at pH 10.2 an equilibrium between forward and reverse reaction determines the final NADH yield, we carefully tested the influence of pH in the range from 10.0 to 11.1 on the initial rate and final NADH yield of the reverse reaction (Figures 4C,D). Between pH 10.0 and pH 10.4, the initial rate was very similar, but the final NADH yield increased steadily. Above pH 10.6 the reaction was considerably slower, and above pH 10.8 a plateau of NADH was not reached within 20 min. We concluded that pH 10.5 ± 0.1 would be the optimal condition for a quantitative assay. Lowering either the NAD^+^ or the enzyme concentration at pH 10.5 progressively slowed down the reaction but had no influence on the amount of NADH formed in the plateau phase (Figures 4E,F). Very similar results were obtained with Arabidopsis P5CR (data not shown). Despite the higher affinity of both P5CR enzymes for NADP^+^, less reliable results were obtained with NADP^+^ as cofactor (not shown), most likely due to the lower reaction rate and the higher pH optimum of the forward reaction (Figure 3E).

**FIGURE 5:**
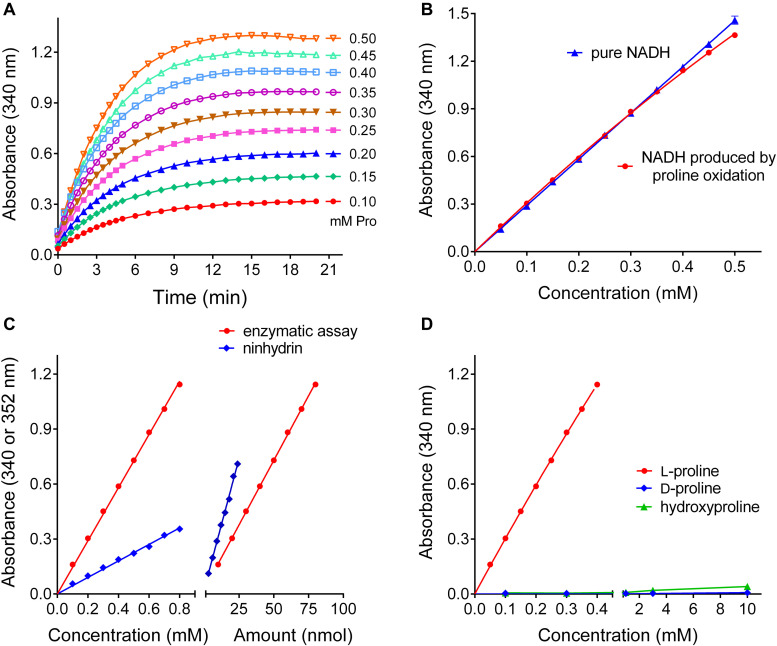
Performance of the optimized P5CR reaction for proline quantification. **(A)** Time- and proline-dependent NADH production by 1 μg rice P5CR at pH 10.5 in the presence of 10 mM NAD^+^ was followed by determining the increase of optical density at 340 nm. **(B)** The comparison of the absorbance of pure NADH and the proline-dependent NADH produced after 15 min under these conditions demonstrated a stoichiometric relationship up to 350 μM proline. **(C)** Sensitivity was compared for the enzymatic assay and the acetate/ninhydrin assay on the basis of either proline concentration, or proline amount in the assayed solution. **(D)** Neither D-proline nor L-hydroxyproline in the micro to millimolar range induced significant NADH formation by P5CR. All data are means ± SE over at least three replicates.

Having determined the optimal conditions for proline quantification with the reverse P5CR reaction, we investigated the range of linearity, sensitivity, and specificity of this method. For proline concentrations between 100 and 500 μM, the plateau of NADH production was consistently reached after 15 min (Figure 5A), and up to 350 μM proline the reaction was virtually complete (Figure 5B). Above this level, the relationship between proline and NADH concentration gradually lost linearity, yet the use of a non-linear fit for absorbance values obtained from known proline concentrations allowed reliable measurement up to 1 mM (data not shown). When the performance of this enzymatic method was compared with the ninhydrin-based assay of Williams and Frank (1975), a similar sensitivity was found. In terms of color development per absolute proline amount, the ninhydrin assay yielded approximately two-fold higher absorbance values (ΔA_352_ = 0.0284 ± 0.0006 nmol^–1^ with ninhydrin vs ΔA_340_ = 0.0141 ± 0.0001 nmol^–1^ for the enzymatic assay) (Figure 5C). However, because the ninhydrin assay uses larger sample volumes, the enzymatic assay is three times more sensitive with respect to proline concentration in the sample (ΔA_340_ = 1.452 ± 0.009 mM^–1^ compared to ΔA_352_ = 0.450 ± 0.006 mM^–1^ with ninhydrin). Concerning specificity, neither D-proline nor hydroxyproline at similar levels induced any NAD^+^ reduction by P5CR (Figure 5D). Similar assay characteristics were obtained with Arabidopsis P5CR, and both enzymes showed no NADH production with P5C, ornithine, pipecolate, or A2CA as substrates (data not shown).

**FIGURE 6:**
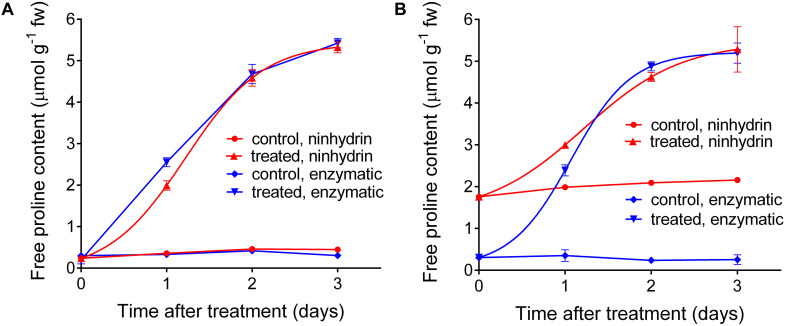
Comparison between the results obtained with the enzymatic and the acidic ninhydrin method according to Williams and Frank (1975). Cell suspension cultures of wild tobacco were treated with 175 mM NaCl. At increasing time after the treatment, culture aliquots were withdrawn and cells were extracted in either 3% (v/v) sulfosalicylic acid **(A)** or in 100 mM glycine-NaOH buffer, pH 10.5 **(B)**. Proline in the two series of extracts was quantified with both the enzymatic and the ninhydrin methods. Data are means ± SE over three biological replicates.

Based on these results, a protocol for the quantitation of free proline in extracts from plant materials was set down (Table 2). This protocol was applied in an experiment in which a *Nicotiana plumbaginifolia* cell culture was treated with salt, and the consequent increase of intracellular free proline was determined at increasing time after the treatment. Cells were also extracted in sulfosalicylic acid and analyzed with the acidic ninhydrin method of Williams and Frank (1975), and the results obtained with the two protocols were compared (Figure 6). When extracts in sulfosalicylic acid were analyzed with either ninhydrin or the enzymatic method, substantially identical results were evident (Figure 6A). A very similar pattern was obtained also with extracts in glycine-NaOH buffer that were analyzed with the enzymatic method. On the contrary, when extracts in glycine-NaOH buffer were analyzed with the ninhydrin method, the amount of proline was overestimated in samples with low proline content, most likely due to the residual presence of proteins (Figure 6B).

**TABLE 2:** Step-by-step protocol for the enzymatic determination of free proline content.

1. Homogenize plant material in 1–5 mL g^–1^ of 100 mM glycine-NaOH buffer, pH 10.5^a^
2. Centrifuge 3–5 min at 10–12,000 *g* at RT, discarding the pellet
3. Treat samples for 5 min at 95°C to inactivate endogenous enzymes
4. In a 96-well plate, put 5–100 μL aliquots of extract in duplicate in a final volume of 100 μL; include reference samples containing 5–100 μL of a standard solution with 1 mM L-Pro
5. Add 100 μL of a pre-warmed 2x reaction mixture containing 10 mM NAD^+^ and 2 μg P5CR in 100 mM glycine-NaOH buffer, pH 10.5 to one of the duplicate samples, to the second sample add 100 μL of an identical mixture without P5CR
6. Follow the increase in absorbance at 340 nm in the P5CR-containing samples until a maximum value is reached (usually 20 to 30 min) and subtract the absorbance value of the samples without P5CR
7. Select the range in which the change in OD_340_ is proportional to the volume of extract and calculate the slope of a regression line
8. Divide the slope of the regression line for a given sample by that of the reference solution: the result represents the concentration of free proline in the extract (in mmol L^–1^); multiply this value for the number of mL extraction buffer g^–1^, to obtain the concentration in μmol g^–1^

*^*a*^As an alternative, plant material can be extracted with 70% ethanol. In this case, following centrifugation, extracts should be brought to dryness at room temperature in a centrifugal vacuum concentrator to remove ethanol, and residues resuspended in 100 mM glycine-NaOH buffer, pH 10.5. This would allow concentration of extracts from samples with very low proline content, and makes step 3 unnecessary.*

## Discussion

A careful analysis of established ninhydrin-based methods for proline quantification revealed that these assays are unreliable, when structurally related compounds or a 30-fold excess of α-amino acids are present in a sample. With our newly developed enzymatic assay, which is specific for L-proline, we present a simple and robust solution for this problem. In extracts from plant cells grown under normo-osmotic conditions, proline typically accounts for only 2–4% of total free amino acids (*e.g.*, Forlani et al., 2013; Giberti et al., 2017). Additionally, depending on the assay conditions, ninhydrin reacts equally well with D-proline or ornithine, but also P5C, hydroxyproline, and pipecolate contribute significantly to the output of the assays. We are not aware of any reports describing the natural occurrence of D-proline in plants, but transporters with the capacity to import D-proline from the growth substrate have been described (Breitkreuz et al., 1999; Lehmann et al., 2011). In contrast, ornithine is both a precursor and a degradation product of arginine (Winter et al., 2015), and in leaves of salt-stressed cashew plants ornithine content reached almost 50% of L-proline concentration (da Rocha et al., 2012). Monteoliva et al. (2014) detected P5C levels below 2 nmol g^–1^ FW in control and pathogen-infected Arabidopsis plants without specifying potential losses of P5C during extraction. By extraction under strongly acidic conditions, which minimizes the loss of P5C prior to analysis, we found that P5C levels are, even after exposure to high concentrations of external proline, undetectable in wild-type Arabidopsis plants, with a detection limit corresponding to 50 nmol g^–1^ FW or 5% of the typical proline content in leaves (Deuschle et al., 2004). Hydroxyproline is typically formed post-translationally by proline hydroxylases and is highly abundant in proline-rich cell wall proteins (Kavi Kishor et al., 2015; Marzol et al., 2018). Free *trans*-4-hydroxy-L-proline was found at low levels in oak and pepper plants and its content changed similarly to L-proline in response to stress (Oufir et al., 2009; del Amor et al., 2010; Florencio-Ortiz et al., 2018). Free *cis*-4-hydroxy-L-proline seems to be a specialty of sandal (*Santalum album* L) and related species, where it constitutes up to 10% of the dry weight in some tissues (Radhakrishnan and Giri, 1954; Kuttan et al., 2015). Pipecolate is a degradation intermediate of lysine and its content was found to be four times higher than L-proline in Arabidopsis leaves after infection with *Pseudomonas syringae* pv *maculicola* (Návarová et al., 2012). Similarly, Thomason et al. (2018) reported an almost 6-fold increase of pipecolate content in heat-stressed wheat leaves. All these examples illustrate the possibility that in several cases the use of the established ninhydrin-based assays would provide inaccurate results and lead to a significant overestimate of proline content and/or an incorrect evaluation of its homeostatic levels under stress.

The results presented in this study show that the reverse reaction of recombinant plant P5CR can be used as a robust and highly specific assay for L-proline. The enzyme that catalyzes proline oxidation under physiological conditions, ProDH, could not be exploited with this aim, since it does not use NAD(P)^+^ as the electron acceptor (Lanfranchi et al., manuscript in preparation). In contrast to previous publications describing the reverse P5CR reaction with NAD^+^ as an assay for quantification of P5CR activity (Chilson et al., 1992; Nocek et al., 2005; Meng et al., 2006), the use for L-proline quantification required slightly more alkaline reaction conditions (pH ≥ 10.5 rather than pH 10 or pH 10.2; Figure 4C). This difference can be explained by the energetics and stoichiometry of the reaction. At pH 7.5, the standard redox potential (E_0_′) of (P5C + 3 H^+^)/proline is −123 mV (Becker and Thomas, 2001), whereas E_0_′ of (NAD^+^ + H^+^)/NADH is −335 mV. Thus, under physiological pH conditions, the reverse reaction is only possible when virtually no NADH is present in the reaction mixture. The asymmetric involvement of protons results in a pH-dependence of the redox potential for (P5C + 3 H^+^)/proline of approximately −90 mV [−2.303 × (RT/*z*F) × *n*, with *z* as the number of transferred electrons and *n* as the number of incorporated protons] per pH unit, yielding −485 mV at pH 10.5. The redox potential of (NAD^+^ + H^+^)/NADH only changes by −30 mV per pH unit, reaching −425 mV at pH 10.5. With a sufficiently large excess of NAD^+^, low concentrations of proline can therefore be quantitatively converted to P5C at this high pH. The slower reaction of P5CR found at pH values exceeding 10.5 (Figure 4D) might depend on a gradual inactivation or denaturation of the enzyme. An additional factor that may promote the reverse reaction at high pH values is the spontaneous equilibrium between cyclic P5C and linear glutamate-5-semialdehyde, which is shifted toward glutamate-5-semialdehyde under alkaline conditions (Mezl and Knox, 1976). According to structural data, only P5C but not glutamate-5-semialdehyde will bind efficiently to the active site of P5CR (Ruszkowski et al., 2015).

Similar to the forward reaction of P5CR, also the reverse reaction was much faster with NAD^+^ than with NADP^+^, and both analyzed enzymes had a higher affinity toward NADP^+^ (Giberti et al., 2014; Forlani et al., 2015a). The additional phosphate group of NADP^+^ is tightly bound by several hydrogen bonds in the crystal structure of *Medicago truncatula* P5CR (Ruszkowski et al., 2015). This stronger binding of NADP^+^ nicely explains both a higher affinity and a slower exchange rate, resulting in the lower maximal reaction rate. Modifications in the structure of the active site of P5CR induced by binding to the phosphate of NADP^+^ may also account for the increased affinity toward proline when NADP^+^ is used as cofactor. Similarly, the affinity of both Arabidopsis and rice P5CR toward P5C was much higher with NADP^+^ as cofactor, which might additionally contribute to the slower reaction rate and inhibition by proline concentrations exceeding 3 and 7 mM, respectively (Giberti et al., 2014; Forlani et al., 2015a). Despite the slower reaction rate, the reverse reaction with NADP^+^ might be useful for the quantification of very low concentrations of proline, although – according to the pH profile of the forward and the reverse reaction – at least pH 11 would be required to eliminate interference from the forward reaction.

We are not aware of any commercial source of plant P5CR, but recombinant expression and affinity purification by FPLC or benchtop columns should be possible in any molecular biology laboratory, and the expression constructs for Arabidopsis or rice P5CR are available upon request. Except a photometer, no special equipment is needed and no harmful chemicals have to be used for this novel assay. When a microplate reader is available, large numbers of samples can be processed in parallel and even automation of the assay would be feasible. By the use of a microvolume photometer, tiny amounts of sample will be sufficient for reliable proline quantification. In contrast to the ninhydrin-based assays, the enzymatic assay operates at moderate temperatures, thereby reducing the risk of volume change by evaporation. We performed all assays at 37°C, but with slightly longer incubation times, also lower temperatures will give reliable results. The sensitivity of the enzymatic assay for proline is very similar to the ninhydrin-based assays and the linear range spans almost one order of magnitude. Therefore, up to a 10-fold increase in proline content can be quantified using identical extraction and measurement conditions. For plants with very high proline content, dilution of the samples may be required. In our hands, a single enzyme preparation from 400 mL of induced *E. coli* culture was enough to perform hundreds of assays with the described protocol. Moreover, the purified enzyme is substantially stable, with less than 10% activity loss after 3 months of storage at 4°C. The source of the enzyme can be selected according to cDNA availability, while the slightly higher *V*_*max*_ of P5CR from Arabidopsis can be exploited to shorten the incubation time. We did not test the reverse reaction of P5CR from other plant species, but we can speculate that they will have similar biochemical and kinetic properties.

In times of non-biased metabolomics, fast and cost-efficient assays to specifically quantify a selected metabolite are still valuable tools for the phenotypic analysis of large populations. Our newly designed assay can be used for both relative and absolute quantification of proline content. In comparison to the traditional, ninhydrin-based assays, it is very similar with respect to sensitivity and dynamic range, but highly superior with respect to specificity.

## Data Availability Statement

The raw data supporting the conclusions of this article will be made available by the authors, without undue reservation.

## Author Contributions

GF designed the study and performed the majority of the experiments. DF prepared the expression plasmids. All authors have contributed to the manuscript and approved its final version.

## Conflict of Interest

The authors declare that the research was conducted in the absence of any commercial or financial relationships that could be construed as a potential conflict of interest.

## Supplementary Material

Supplementary Figure 1 Affinity purification of heterologously expressed rice and Arabidopsis δ^1^-pyrroline-5-carboxylate reductase (P5CR). 6×-His-tagged P5CRs from Arabidopsis (32.5 kDa) and rice (33.5 kDa) were expressed in *Escherichia coli* as previously described (Giberti et al., 2014; Forlani et al., 2015a). Soluble protein extracts from IPTG-induced cells were loaded onto His SpinTrap columns (GE Healthcare) and bound proteins were eluted with increasing concentrations of imidazole, as indicated. Proteins were determined by the method of Bradford (1976), and activity was measured as the P5C-dependent oxidation of NADPH. Selected fractions (E, crude extract; ft, flow through) were analyzed by SDS-PAGE (12% separating gel) and Coomassie staining. The arrows indicate the position of the recombinant enzymes in the crude extracts. ^∗^, molecular weight markers.

Supplementary Figure 2 Kinetic analysis of the reverse reaction of rice and Arabidopsis P5C reductase (P5CR). The purified enzymes (25 and 50 ng for Arabidopsis and rice P5CR, respectively) were incubated at 37°C in 100 mM glycine-NaOH buffer, pH 10.2. The proline-dependent reduction of NAD(P)^+^ was measured photometrically at 340 nm. The invariable substrates were fixed at 10 mM NADP^+^ or 20 mM NAD^+^ **(A)**; 50 mM (with NAD^+^) or 5 mM (with NADP^+^) proline **(B)**; 3 mM NADP^+^ or 15 mM NAD^+^ **(C)**; 2 mM proline **(D)**. One unit of enzyme activity is herein defined as the corresponding forward reaction rate measured at pH 7.5 under saturating substrate conditions (1 mM L-P5C and 0.4 mM NADPH). Data are mean ± SE over three replicates.
