# Supplementary material for: A Specific and Sensitive Enzymatic Assay for the Quantitation of L-Proline
Source: Front Plant Sci. 2020 Oct 22;11:582026. doi: 10.3389/fpls.2020.582026 (PMC7642206; doi:10.3389/fpls.2020.582026)
Supplement: Supplementary Figure 1 — Affinity purification of heterologously expressed rice and Arabidopsis δ1-pyrroline-5-carboxylate reductase (P5CR). 6×-His-tagged P5CRs from Arabidopsis (32.5 kDa) and rice (33.5 kDa) were expressed in Escherichia coli as previously described (Giberti et al., 2014; Forlani et al., 2015a). Soluble protein extracts from IPTG-induced cells were loaded onto His SpinTrap columns (GE Healthcare) and bound proteins were eluted with increasing concentrations of imidazole, as indicated. Proteins were determined by the method of Bradford (1976), and activity was measured as the P5C-dependent oxidation of NADPH. Selected fractions (E, crude extract; ft, flow through) were analyzed by SDS-PAGE (12% separating gel) and Coomassie staining. The arrows indicate the position of the recombinant enzymes in the crude extracts. ∗, molecular weight markers. [file Data_Sheet_1.PDF]

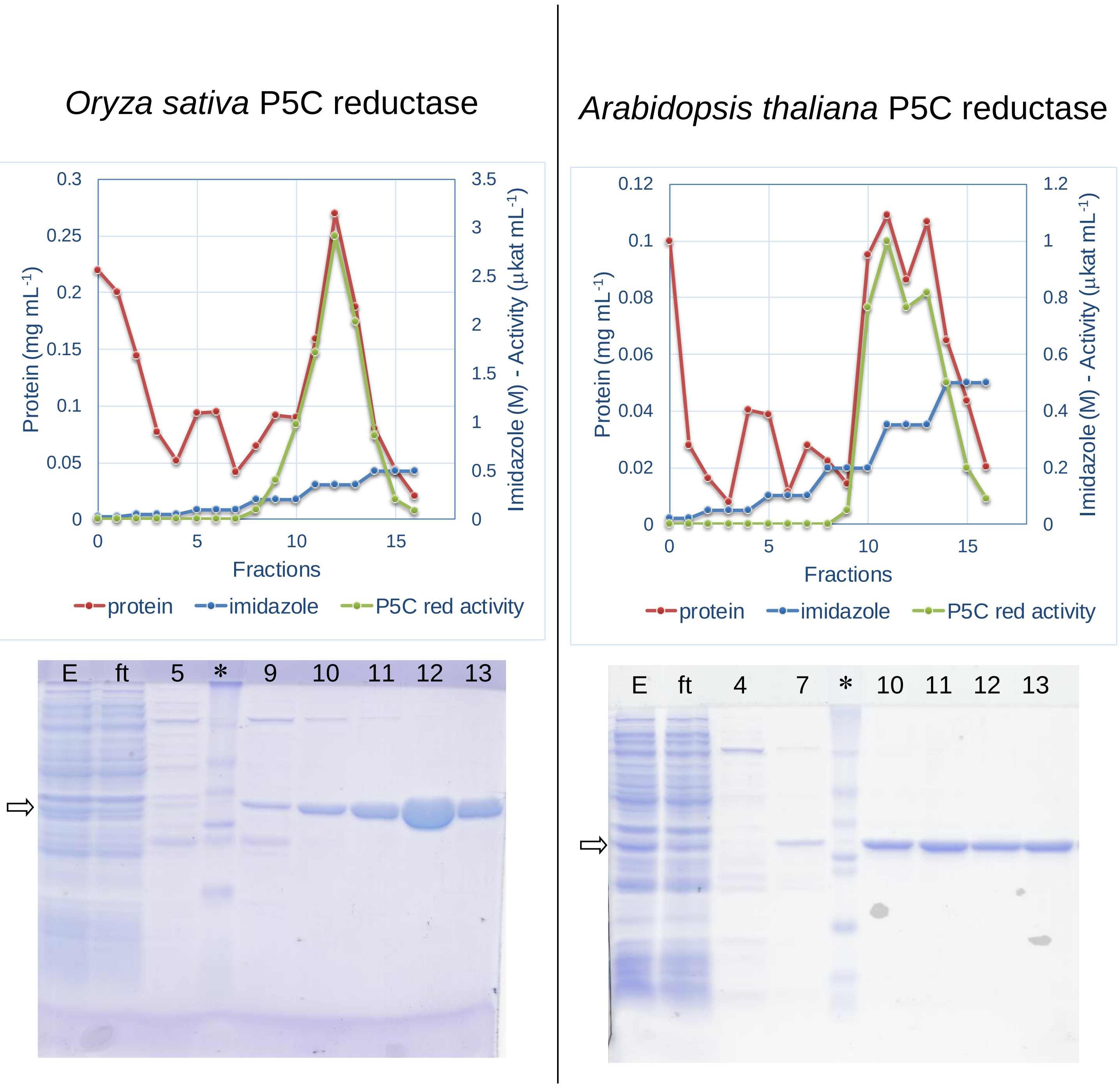

Figure S1 Affinity purification of heterologously expressed rice and Arabidopsis δ^1-pyrroline-5-carboxylate reductase (P5CR). 6x-His-tagged P5CRs from Arabidopsis (32.5 kDa) and rice (33.5 kDa) were expressed in *Escherichia coli* as previously described (Giberti et al., 2014; Forlani et al., 2015). Soluble protein extracts from IPTG-induced cells were loaded onto His SpinTrap columns (GE Healthcare) and bound proteins were eluted with increasing concentrations of imidazole, as indicated. Proteins were determined by the method of Bradford (1976), and activity was measured as the P5C-dependent oxidation of NADPH. Selected fractions (E, crude extract; ft, flow through) were analyzed by SDS-PAGE (12% separating gel) and Coomassie staining. The arrows indicate the position of the recombinant enzymes in the crude extracts. *, molecular weight markers.

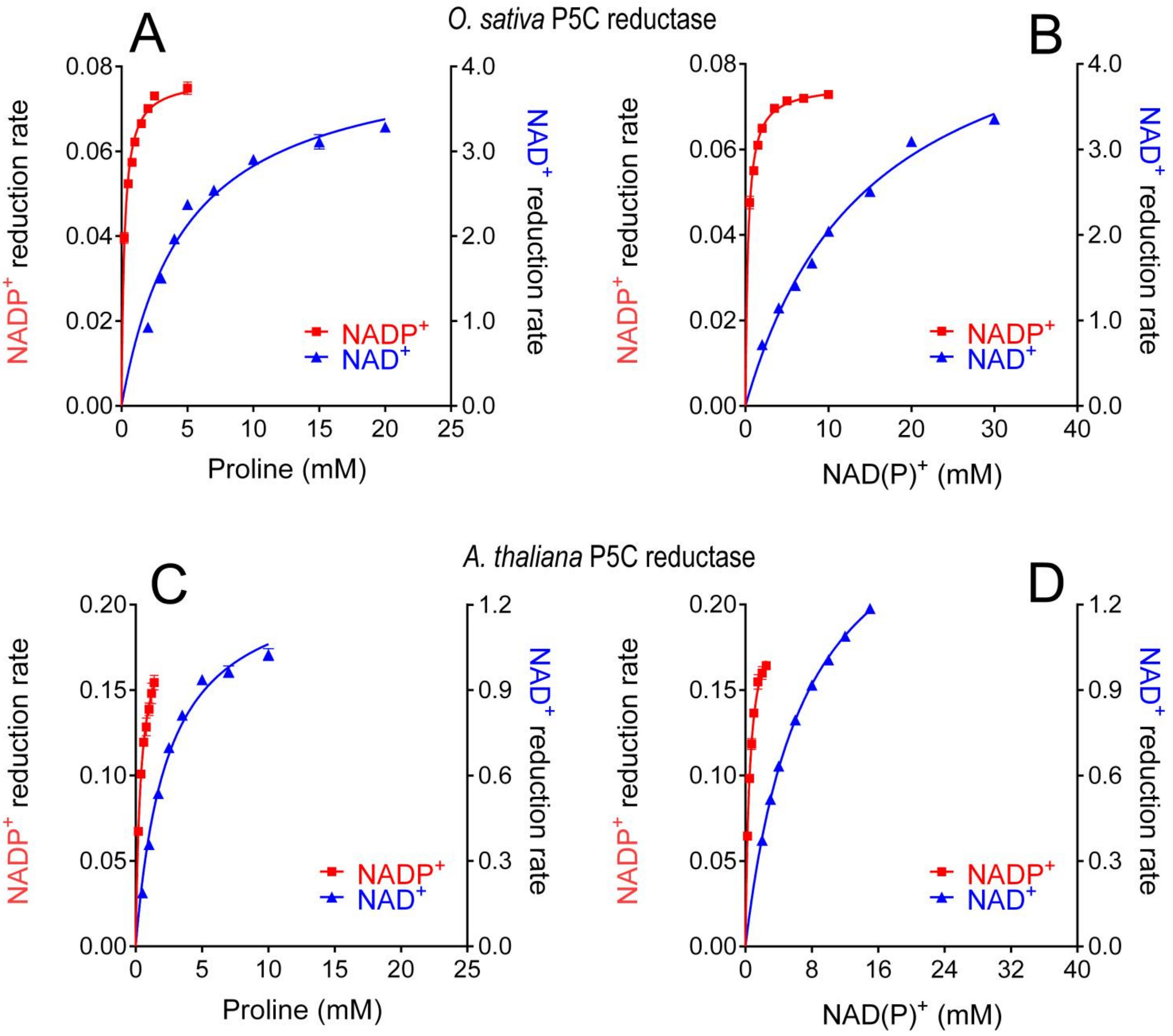

Figure S2 Kinetic analysis of the reverse reaction of rice and Arabidopsis P5C reductase (P5CR). The purified enzymes (25 and 50 ng for Arabidopsis and rice P5CR, respectively) were incubated at 37°C in 100 mM glycine-NaOH buffer, pH 10.2. The proline-dependent reduction of $NAD(P)^+$ was measured photometrically at 340 nm. The invariable substrates were fixed at 10 mM $NADP^+$ or 20 mM NAD^+ (A); 50 mM (with NAD^+) or 5 mM (with $NADP^+$) proline (B); 3 mM $NADP^+$ or 15 mM NAD^+ (C); 2 mM proline (D). One unit of enzyme activity is herein defined as the corresponding forward reaction rate measured at pH 7.5 under saturating substrate conditions (1 mM L-P5C and 0.4 mM NADPH). Data are mean ± SE over three replicates.
